# Biomarkers of Cellular Senescence and Skin Aging

**DOI:** 10.3389/fgene.2018.00247

**Published:** 2018-08-23

**Authors:** Audrey S. Wang, Oliver Dreesen

**Affiliations:** ^1^Cell Ageing, Skin Research Institute of Singapore (SRIS), A^∗^STAR, Singapore, Singapore; ^2^Lee Kong Chian School of Medicine, Nanyang Technological University, Singapore, Singapore

**Keywords:** senescence, skin, aging, biomarkers, photoaging, cancer

## Abstract

Cellular senescence is an irreversible growth arrest that occurs as a result of different damaging stimuli, including DNA damage, telomere shortening and dysfunction or oncogenic stress. Senescent cells exert a pleotropic effect on development, tissue aging and regeneration, inflammation, wound healing and tumor suppression. Strategies to remove senescent cells from aging tissues or preneoplastic lesions can delay tissue dysfunction and lead to increased healthspan. However, a significant hurdle in the aging field has been the identification of a universal biomarker that facilitates the unequivocal detection and quantification of senescent cell types *in vitro* and *in vivo*. Mammalian skin is the largest organ of the human body and consists of different cell types and compartments. Skin provides a physical barrier against harmful microbes, toxins, and protects us from ultraviolet radiation. Increasing evidence suggests that senescent cells accumulate in chronologically aged and photoaged skin; and may contribute to age-related skin changes and pathologies. Here, we highlight current biomarkers to detect senescent cells and review their utility in the context of skin aging. In particular, we discuss the efficacy of biomarkers to detect senescence within different skin compartments and cell types, and how they may contribute to myriad manifestations of skin aging and age-related skin pathologies.

## Cellular Senescence and Organismal Aging

Aging is the result of a gradual functional decline at the cellular, and ultimately, organismal level, resulting in the development of myriad chronic illnesses including heart disease, stroke and diabetes. On a cellular level, aging was first described by Hayflick and Moorhead (1961), who demonstrated that somatic mammalian cells have a finite propensity for cell division, after which they enter an irreversible growth arrest termed cellular senescence. Senescent cells are characterized by their inability to proliferate, resistance to apoptosis, and secretion of factors that promote inflammation and tissue deterioration (Campisi and d’Adda di Fagagna, 2007; Kuilman et al., 2010; He and Sharpless, 2017). The molecular basis underlying Hayflick’s observations came to light only decades later. In the early 1970s, Watson and Olovnikov proposed that during semi-conservative DNA replication of linear chromosomes, the removal of the terminal RNA primer, required to initiate lagging strand synthesis, would result in a gap that can no longer be filled by conventional DNA polymerases. As a result of this “end replication problem", chromosome terminal sequences, termed telomeres (Blackburn and Challoner, 1984) shorten during each replication cycle (Hayflick and Moorhead, 1961; Watson, 1972; Olovnikov, 1973; Allsopp et al., 1992). Critically shortened telomeres trigger a persistent activation of DNA damage response (DDR) pathways, thereby resulting in cellular senescence (D’Adda Di Fagagna et al., 2003). It is now clear that several types of cellular stressors can trigger senescence. These include telomere shortening and dysfunction (D’Adda Di Fagagna et al., 2003; Takai et al., 2003), inadvertent activation of oncogenes, termed oncogene-induced senescence (OIS) (Serrano et al., 1997; Michaloglou et al., 2005; Suram et al., 2012), changes in chromatin structure and epigenetic stress (Bakkenist and Kastan, 2003; Munro et al., 2004), oxidative stress, mitochondrial dysfunction (Wiley et al., 2016), or persistent activation of DNA damage checkpoints (D’Adda Di Fagagna et al., 2003; Rodier et al., 2009). There is increasing evidence suggesting that senescent cells accumulate in aging tissues and organs, thereby impairing physiological processes, including regeneration, and contributing to organismal aging (Dimri et al., 1995; Krishnamurthy et al., 2004; Jeyapalan et al., 2007).

## Senescence Biomarkers

The identification of unique markers that unequivocally detect and quantify senescent cells, particularly *in vivo*, remains challenging (Sharpless and Sherr, 2015). Senescent cells display an enlarged and flattened cell shape (Hayflick, 1965; Chen et al., 2000, 2008), and elevated senescence-associated β-galactosidase (SA-β-gal) activity, which remains the gold standard to identify senescent cells in culture and tissue samples (Dimri et al., 1995; Debacq-Chainiaux et al., 2009). However, this assay requires active enzymatic SA-β-gal activity, which is retained in fresh tissue samples but often lost in fixed or cryopreserved tissues (Severino et al., 2000; Lee et al., 2006). In addition, non-specific SA-β-gal activity can be detected in early passage adult melanocytes proliferating in culture, in hair follicles, sebaceous glands, and eccrine glands and ducts in both young and old human skin (Dimri et al., 1995), as well as in proliferating surface luminal cells of the duodenum (Going et al., 2002). Importantly, it remains difficult to multiplex the conventional detection method for SA-β-gal with other senescence-associated or cell-type specific markers, hence limiting the ability to identify senescent cell types or populations within a complex tissue or organ. Toward this goal, a modified flow cytometry-based method of multiplexing SA-β-gal with other senescence markers has been reported (Biran et al., 2017). However, such multiplexing strategies involving SA-β-gal in tissue biopsies *in situ* remains laborious. Despite being a hallmark of senescent cells, SA-β-gal activity does not play a mechanistic role in triggering senescence: cells from patients with autosomal recessive G(M1)-gangliosidosis lack lysosomal β-gal activity, but retain their ability to senesce (Lee et al., 2006).

As senescent cells are terminally growth arrested, cell cycle regulators such as p16^INK4a^, p21^CIP1^ and p53 (Collado and Serrano, 2010) are commonly employed to detect senescent cells. p16^INK4a^, p21^CIP1^ and p53 were simultaneously upregulated in human skin fibroblasts post-ultraviolet (UV) radiation (Chen et al., 2008). p16^INK4a^ plays a key role in cell cycle control upstream of the retinoblastoma tumor suppressor protein, and p16^INK4a^-positive senescent cells accumulate in an age-dependent manner in multiple tissues including the skin (Zindy et al., 1997; Krishnamurthy et al., 2004; Michaloglou et al., 2005; Herbig et al., 2006; Ressler et al., 2006; Coppé et al., 2011; Waaijer et al., 2012). p16^INK4a^-positive cells accumulate in preneoplastic lesions, including melanocyte-rich benign human nevi, caused by activating mutations in N-RAS or its downstream target BRAF (Michaloglou et al., 2005; Ivanov et al., 2013). Considering the role of p16^INK4a^ in mediating senescence, it is not surprising that this locus is frequently mutated in a variety of human cancers, including skin epithelial tumors (Soufir et al., 1999; De Snoo et al., 2008).

Although less well understood, senescence is also characterized by widespread chromatin remodeling. Normal cellular aging is associated with global heterochromatin loss, characterized by markers H3K9me3 and H3K27me3 (Tsurumi and Li, 2012). In agreement with these findings, cells from patients with the accelerated aging syndrome Hutchinson–Gilford progeria syndrome (HGPS) exhibit a profound loss of heterochromatin (Misteli and Scaffidi, 2005; Shumaker et al., 2006; Chojnowski et al., 2015). Predominantly during OIS *in vitro*, heterochromatin is redistributed into 30–50 punctate DNA-dense senescence-associated heterochromatin foci (SAHF). SAHF are silent domains that co-localize with H3K9me3 and heterochromatin protein 1 (HP1) and may lock cells in a senescent state by transcriptionally repressing genes involved in cell proliferation (Narita et al., 2003; Sadaie et al., 2013; Shah et al., 2013). In contrast, SAHF formation was not observed in HGPS patient cells or preneoplastic lesions that express other senescence markers (Scaffidi and Misteli, 2006; Shumaker et al., 2006; Kosar et al., 2011; Ivanov et al., 2013). Long-term monitoring of senescent cells *in vitro* revealed progressive proteolysis of Histones 3 and 4 without DNA loss. Reduced histone content was also observed in nevus melanocytes, as compared to neighboring non-senescent melanocytes and keratinocytes *in vivo* (Ivanov et al., 2013). These studies confirm the dramatic structural changes of chromatin in senescent cells. In addition, the same authors also noted the presence of cytoplasmic chromatin fragments (CCFs) in ∼20% of cells undergoing replicative senescence (RS) or OIS *in vitro*. CCFs were positive for H3K9me3 and γ-H2A-X, but negative for 53BP1 and lamin A/C, and targeted for degradation by autophagy. The accumulation of such chromatin fragments within the cytoplasm suggests that the integrity of the nuclear envelope becomes compromised in senescent cells (Ivanov et al., 2013).

Indeed, the nuclear lamina, a proteinaceous network that lies beneath the inner nuclear membrane, undergoes dramatic remodeling during cellular senescence (Shimi et al., 2011; Freund et al., 2012; Dreesen et al., 2013a). Lamin B1, an intermediate filament protein expressed in all somatic cells (Stewart and Burke, 1987; Hoger et al., 1988), is downregulated in cells undergoing RS, OIS and UV-induced senescence *in vitro* (Shimi et al., 2011; Freund et al., 2012; Dreesen et al., 2013a; Ivanov et al., 2013; Sadaie et al., 2013; Shah et al., 2013; Wang et al., 2017). Lamin B1 levels also decline during chronological aging of human skin *in vivo* (Dreesen et al., 2013a,b), in senescent melanocytes within human nevi (Ivanov et al., 2013), in UV-exposed mouse skin epidermis (Wang et al., 2017), irradiated mouse liver (Freund et al., 2012) and in kidneys of a premature aging mouse model (Baar et al., 2017). Importantly, by co-staining with cell-type specific markers, lamin B1 staining facilitated the identification and quantification of senescent melanocytes within nevi as compared to neighboring keratinocytes within the epidermis (Ivanov et al., 2013). Similarly, co-staining lamin B1 with a keratinocyte differentiation marker followed by single cell analysis enabled us to quantify the accumulation and clearance of senescent cells in different epidermal compartments after UV exposure and upon regeneration, respectively (Wang et al., 2017). In addition to lamin B1, the inner nuclear membrane protein lamin B receptor (LBR) and the lamina-associated polypeptide-α (LAP2α) are also downregulated in senescent cells (Dreesen et al., 2013a; Ivanov et al., 2013; Lukášová et al., 2017). However, LBR levels vary within the different epidermal layers in mouse skin, indicating that its expression may be altered during keratinocyte differentiation (Solovei et al., 2013). Hence, this may limit the usage of LBR as an adequate senescence marker in human skin. Moreover, loss of LAP2α is not specific to senescent cells and also occurs in quiescent cells (Pekovic et al., 2007; Dreesen et al., 2013a). Thus, co-staining of lamin B1 and LAP2α distinguishes senescent from quiescent cells (Dreesen et al., 2013a). The mechanism of senescence-induced lamin B1 downregulation has been elucidated in detail by several groups. *LMNB1* transcription is decreased (Shimi et al., 2011; Freund et al., 2012; Dreesen et al., 2013a) and *LMNB1* mRNA is destabilized, possibly via targeted degradation by miR-23a (Lin and Fu, 2009; Dreesen et al., 2013a). Additionally, lamin B1 protein is shuttled to the cytoplasm and degraded via autophagy (Dou et al., 2015). It should be noted that *LMNB1* mRNA levels alone are not a reliable indicator of senescence. For instance, *LMNB1* mRNA levels decrease in contact-inhibited quiescent fibroblasts, whereas lamin B1 protein levels remain stable as cells do not turn over (Dreesen et al., 2013a). Taken together, although the mechanistic role of lamin B1 during cellular senescence is not fully understood, loss of lamin B1 is emerging as a potentially important biomarker to identify and quantify senescent cells *in vitro* and *in vivo*.

Concomitant with senescence-associated loss in chromatin organization, overall DNA methylation and expression of the DNA methyl-transferase DNMT1 decrease during cellular aging. These altered DNA methylation signatures can distinguish cells based on their developmental potential and may be used to predict chronological and biological age (Horvath, 2013; Raddatz et al., 2013). Such distinct methylation patterns are also associated with chronologically aged and photoaged skin (Grönniger et al., 2010).

Persistent DNA damage is a critical trigger of cellular senescence and can be identified by the presence of γ-H2A-X and 53BP1 foci (D’Adda Di Fagagna et al., 2003) and activated ataxia-telangiectasia mutated (ATM) kinase (Zou, 2007). However, while DNA damage *per se*, is not a marker for cellular senescence, the occurrence of telomere-associated DNA damage foci (TAF) has been used to detect senescent cells and quantify tissue aging (Herbig et al., 2006; Jeyapalan et al., 2007; Hewitt et al., 2012; Waaijer et al., 2018). Replicative senescent baboon skin fibroblasts and skin biopsies from aged baboons displayed high incidence of TAF as indicated by co-localization of 53BP1 and γ-H2A-X on telomeric DNA (Herbig et al., 2006; Jeyapalan et al., 2007). In agreement with these findings, shortened telomeres have been detected in skin from aged individuals, in sun-exposed skin and premalignant skin lesions including actinic keratosis (AK) (Sugimoto et al., 2006; Ikeda et al., 2014). Other age-related genomic changes occur in mitochondrial DNA (mtDNA): age-dependent increase of mtDNA mutations and a common 4977 bp mtDNA deletion have been reported in several human tissues and cell types (Passos et al., 2007; Wiley et al., 2016), as well as in photoaged human skin (Berneburg et al., 1997; Koch et al., 2001). Lastly, a histone variant H2A.J accumulates in an age-dependent manner in mouse and human skin, and in irradiated mouse skin. Both H2A.J and p16^INK4a^ levels were elevated in carcinogen-induced preneoplastic lesions but decreased in more advanced papillomas in mouse skin. H2A.J upregulation promotes expression of inflammatory genes, further exacerbating the senescence-induced hyperinflammation (Contrepois et al., 2017).

For long, senescent cells were considered passive bystanders that ceased to proliferate. Research in the past decade revealed that senescence can dramatically change cell function. For instance, senescent cells modulate their environment by secreting inflammatory cytokines, chemokines, matrix metalloproteinases (MMPs) and growth factors, collectively known as the senescence-associated secretory phenotype (SASP) (Acosta et al., 2008; Coppé et al., 2008b; Kuilman et al., 2008; Rodier et al., 2009) or senescence-messaging secretome (SMS) (Kuilman and Peeper, 2009). As such, the presence of MMPs (e.g., MMP3 and MMP9), chemokines (e.g., CXCR2), cytokines (e.g., IL-6 and IL-8) (Acosta et al., 2008; Kuilman et al., 2008) and insulin-like growth factor binding protein 7 (IGFBP7) (Wajapeyee et al., 2008) has been used as markers for senescent dermal fibroblasts and melanocytes *in vitro*. *In vivo*, elevated IL-6 has been detected in nevi melanocytes (Ghosh and Capell, 2016) while MMPs are detected in chronologically aged and photoaged skin, and are responsible for breakdown of the extracellular matrix (ECM) (Quan et al., 2009; Quan and Fisher, 2015). In addition, the plasminogen activator inhibitor type 1 (PAI-1), an inhibitor of serine proteases, is elevated in dermal fibroblasts derived from patients with the premature aging syndromes HGPS and Werner (Murano et al., 1991; Goldstein et al., 1994; Chojnowski et al., 2015), chronologically aged donors (Goldstein et al., 1994), and in tissues of premature aging BubR1 mutant mice (Baker et al., 2008, 2011). PAI-1 plays a role in triggering senescence downstream of p53 (Kortlever et al., 2006) and is both a mediator and marker of senescence. Interestingly, most studies focused on fibroblasts *in vitro* and it remains to be determined whether the secretome of other senescent cell types differs – and how this may affect the function of neighboring cells. It is noteworthy that expression of p53 or p16^INK4a^ alone may not be sufficient to trigger a SASP as a persistent DDR is mandatory (Rodier et al., 2009; Coppé et al., 2011).

High mobility group box-1 (HMGB1) belongs to the family of alarmins, which is an inflammatory mediator important in tissue damage signaling (Bianchi, 2006; Yamada and Maruyama, 2007). In senescent cells, HMGB1 translocates from the nucleus to the cytoplasm and extracellular space, facilitating the release of SASP factors including IL-1β, IL-6 and MMP3 (Davalos et al., 2013; Biran et al., 2017). UVB-exposure stimulated the release of HMGB1, IL-1 and IL-6 in human keratinocytes *in vitro*, and reduced nuclear Hmgb1 expression in mouse skin epidermis *in vivo* (Johnson et al., 2013). Although HMGB1 is used as a marker to detect senescent cells in various contexts (Davalos et al., 2013; Wiley et al., 2016; Biran et al., 2017), its utility to detect senescent cells in human skin remains to be investigated.

**FIGURE 1:**
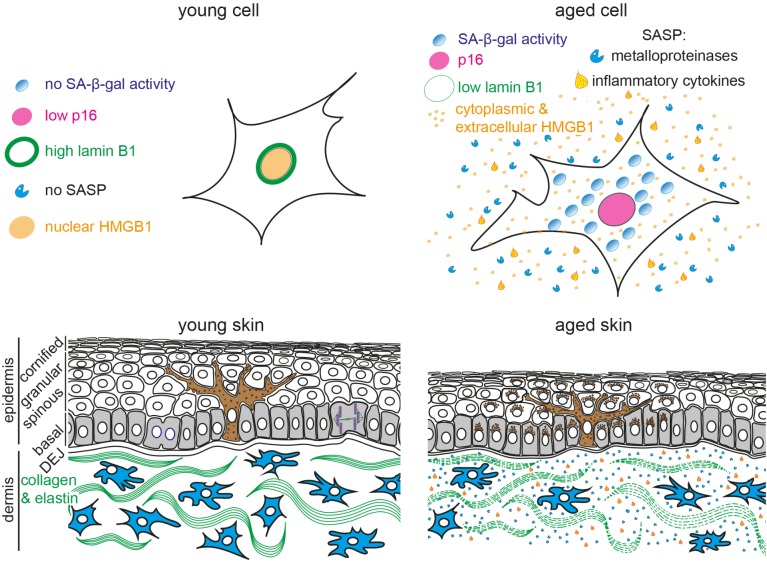
Schematic representation of young and aged human skin. **(Top)** Illustration of young **(Left)** and aged senescent cells **(Right)**. Senescent cells are characterized by (1) enlarged and flattened cell morphology, (2) increased senescence-associated β-galactosidase (SA-β-gal) activity, (3) p16 upregulation, (4) reduced lamin B1 expression, (5) translocation of nuclear HMGB1 into the cytoplasm and extracellular space and (6) secretion of senescence-associated secretory phenotype (SASP) factors, including inflammatory cytokines and metalloproteinases. **(Bottom)** The epidermis consists of keratinocytes, arranged into basal (gray), spinous, granular and cornified layers. Keratinocytes progressively flatten as they move apically and lose their nuclei. Melanocytes (brown) reside within the basal layer; their dendrites branch to neighboring keratinocytes to facilitate pigment transfer. The dermal-epidermal junction (DEJ) separates the epidermis from the underlying dermis. Dermal fibroblasts (blue) reside among collagen and elastin fibers (green fibers) in the dermis. Aged skin **(Right)** becomes atrophic due to reduced proliferation, exhibits abnormal pigmentation, increased inflammation and breakdown of collagen fibers.

Taken together, various senescence markers are currently being used individually and in combination, in different biological contexts *in vitro* and *in vivo* (**Figure 1** and **Table 1**). Nevertheless, further characterization of current markers and the identification of additional markers are indispensable to reliably detect and quantify senescent cells *in vitro* and *in vivo*. In particular, it is essential to develop better methods to multiplex senescence markers with other cell-type specific markers to identify and quantify senescent cell types in multi-compartment organs such as the skin and its appendages including sebaceous glands and hair follicles. Here, we will focus on what is known about the accumulation of senescent cells in skin, how they may affect skin function and contribute to age-related skin pathologies.

**Table 1:** Biomarkers previously described in skin aging.

Biomarkers	*In vitro*	*In vivo*	Reference
SA-β-gal	RS fibroblasts, keratinocytes, melanocytes (*Hu*), UVI keratinocytes and fibroblasts (*Hu*)	CA epidermis (*Hu*) UVI epidermis (*Ms*)	Dimri et al., 1995 Chen et al., 2008; Wang et al., 2017
Enlarged, flattened cell morphology	RS fibroblasts (*Hu, Bb*), UVI keratinocytes and fibroblasts (*Hu*)	Not well documented	Hayflick and Moorhead, 1961; Chen et al., 2000, 2008; Jeyapalan et al., 2007; Wang et al., 2017
p16^INK4a^	OIS melanocytes (*Hu*), RS fibroblasts (*Bb*) and ROT-treated fibroblasts (*Hu*)	Nevi melanocytes (*Hu*), premalignant papillomas (*Ms*), CA skin (*Bb*), CA epidermis, dermis and hair follicles (*Hu*)	Collado et al., 2005; Michaloglou et al., 2005; Herbig et al., 2006; Ressler et al., 2006; Jeyapalan et al., 2007; Waaijer et al., 2012, 2018; Ivanov et al., 2013
Lamin B1	UVI keratinocytes (*Hu*) and OIS melanocytes (*Hu*)	CA epidermis (*Hu*), nevi melanocytes (*Hu*) and UVI epidermis (*Ms*)	Dreesen et al., 2013a,b; Ivanov et al., 2013; Wang et al., 2017
SASP (cytokines, MMPs, growth factors)	OIS melanocytes (IL-1, IL-6, IL8, IGFBP7; *Hu*), CA fibroblasts (IL-6, IL-8; *Hu*), UVI keratinocytes (IL-1, IL-6, HMGB1; *Hu*), HGPS and WS fibroblasts (PAI-1; *Hu*)	CA and UVI epidermis (MMPs, *Hu*), UVI skin (Hmgb1; *Ms*), nevi melanocytes (IL-6; *Hu*), and DNA damaged fibroblasts (IL-6, IL-8, Mmp1, Mmp3; *Ms*)	Murano et al., 1991; Goldstein et al., 1994; Coppé et al., 2008b; Kuilman et al., 2008; Wajapeyee et al., 2008; Jun and Lau, 2010; Johnson et al., 2013; Chojnowski et al., 2015; Quan and Fisher, 2015; Ghosh and Capell, 2016
Telomere-associated foci (TAFs)	RS fibroblasts (*Bb*) and ROT-treated fibroblasts (*Hu*)	CA skin (*Bb*)	Herbig et al., 2006; Jeyapalan et al., 2007; Waaijer et al., 2018
mtDNA modification	UVI keratinocytes (*Hu*)	UVI skin (*Hu*)	Berneburg et al., 1997; Koch et al., 2001
Chromatin modification	HGPS fibroblasts (H3K27me3; *Hu*) and OIS melanocytes (CCFs, H3, H4; *Hu*)	Nevi melanocytes (H3; *Hu*), CA skin (H2A.J; *Ms, Hu*), UVI and premalignant lesions (H2A.J; *Ms*)	Ivanov et al., 2013; Chojnowski et al., 2015; Contrepois et al., 2017


*Hu, human; Ms, mouse; Bb, baboon; SA-β-gal, senescence-associated β-galactosidase; UVI, ultraviolet irradiated; RS, replicative senescent; OIS, oncogene-induced senescent; ROT, rotenone; CA, chronologically aged; SASP, senescence-associated secretory phenotype; MMPs, matrix metalloproteinases; mtDNA, mitochondria DNA; CCFs, cytoplasmic chromatin fragments; H3, histone 3; H4, histone 4.*

## Skin Aging

Skin is the largest organ of the human body: it provides a barrier against harmful organisms and substances, protects against UV radiation and regulates water loss and body temperature (Blanpain and Fuchs, 2006). The skin is a complex organ, consisting of several compartments with different functions. The outermost epidermis is stratified into four sublayers (basal, spinous, granular and cornified layer) with keratinocytes being the predominant cell type. Pigment-producing melanocytes reside within the basal layer of the epidermis, determine skin color and possess photo-protecting properties (**Figure 1**). The dermal-epidermal junction (DEJ) connects the epidermis to the underlying dermis, which harbors dermal fibroblasts and appendages such as hair follicles, sebaceous glands, and sweat glands.

Skin aging is a multi-factorial process that affects nearly every aspect of its biology and function; it is driven by both intrinsic (e.g., time, genetic factors, hormones) and extrinsic (e.g., UV exposure, pollution) factors. Characteristics of intrinsic or chronological aging include visible signs such as thin and dry skin, fine wrinkles, decreased elasticity, aberrant pigmentation, hair graying and hair loss. Epidermal thinning is, in part, caused by decreased proliferation and renewal capacity of basal keratinocytes and reduced epidermal stem cell number (Kligman, 1979; Montagna and Carlisle, 1979; Mimeault and Batra, 2010; Dreesen et al., 2013a; López-Otín et al., 2013). Besides the epidermis, both the DEJ and dermis also become thinner (Lavker et al., 1986). Fibroblasts residing within the dermis generate an ECM that provides the skin with structural integrity and elasticity. During aging, the ECM undergoes structural alterations and degradation (**Figure 1**), thought to result in dermal thinning, increased wrinkling and loss of elasticity (Shuster et al., 1975). Although the number of senescent cells increases during chronological aging of human skin (Dimri et al., 1995; Ressler et al., 2006; Waaijer et al., 2012; Dreesen et al., 2013a; Ghosh and Capell, 2016), a more detailed characterization as to which cell types or skin compartment undergoes senescence is necessary.

## UV and Skin Aging

In addition to chronological aging, extrinsic factors including exposure to sunlight, pollutants and cigarette smoke can accelerate skin aging. While most UVC is blocked by the ozone layer, UVA and UVB rays reach the earth’s surface and contribute to skin aging and cancer development. UVA is a weak mutagen, yet penetrates into the dermis contributing to oxidative stress and tissue inflammation. UVB is more mutagenic as it directly interacts with DNA to generate dipyrimidine photoproducts, resulting in DNA damage during DNA replication (Ravanat et al., 2001; You et al., 2001). Acute UV radiation results in sunburns, aberrant pigmentation and immune suppression, whilst chronic exposure is associated with premature skin aging and a predisposition to develop malignancies (de Gruijl, 1999; Ichihashi et al., 2003; Quan et al., 2009; Behrens et al., 2014; Rittié and Fisher, 2015). Sun-exposed skin appears thick and rough, marked with coarse wrinkles, visible appearance of blood vessels under the skin surface (telangiectasia) and aberrant pigmentation (Ma et al., 2001; Wlaschek et al., 2001). A key histological hallmark of photodamaged skin is the accumulation of amorphous elastic fibers (solar elastosis), accompanied by fragmented and disorganized collagen in the dermis (Varani et al., 2004, 2006; Quan et al., 2009; Watson et al., 2014). Solar elastosis may be a consequence of impaired elastic and fibrillin production, elevated breakdown by MMPs secreted by senescent cells, or a direct consequence of UV exposure (Sherratt, 2013; Quan and Fisher, 2015; Pittayapruek et al., 2016). *In vitro*, UVB-exposed skin cell types (fibroblasts, keratinocytes) exhibit DNA damage, cell cycle arrest and express senescence biomarkers such as increased SA-β-gal activity, p16^INK4a^, p21^CIP1^, p53 activation and lamin B1 downregulation (Chainiaux et al., 2002; Debacq-Chainiaux et al., 2005; Lewis et al., 2008; McCart et al., 2017; Wang et al., 2017). *In vivo*, chronic low dose exposure to UVB resulted in accumulation of DNA damage and lamin B1-low senescent cells within the mouse epidermis, but not the dermis (Wang et al., 2017), a reduction in stem cell numbers in the hair follicle, and p21^CIP1^ accumulation in epidermis and hair follicle stem cell region (McCart et al., 2017).

Although the number of melanocytes in our skin decreases and skin color in sun-protected areas lightens with age (Gilchrest et al., 1979; Ortonne, 1990), aberrant pigmentation (hyper- and hypopigmentation; i.e., sun spots and freckles) occurs at chronically sun-exposed body parts (Ortonne, 1990; Chung, 2003). Interestingly, in a 3D organotypic skin model comprised of keratinocytes, melanocytes and fibroblasts, the presence of aged fibroblasts resulted in increased melanogenic gene transcription, increased epidermal melanin and hyperpigmentation (Duval et al., 2014). Diverse pigmentary changes occur in aged skin and future studies will investigate whether senescent cell types accumulate at these lesions and how they may affect pigmentation. We anticipate that multiplexing senescence markers with cell-type specific markers will shed light as to how chronological aging and/or UV exposure modulate the complex interplay between pigment-producing melanocytes and their neighboring epidermal keratinocytes and dermal fibroblasts.

## Pollution and Skin Aging

More recently, it has been shown that air pollution contributes to various aspects of skin aging. Residents living near highly polluted areas and exposed to high levels of traffic-related particles and nitrogen dioxide developed pigmentary changes and increased coarse wrinkles (Vierkötter et al., 2010; Hüls et al., 2016). Similarly, exposure to tobacco smoke accelerates skin aging, resulting in increased wrinkling, elastosis, telangiectasia and laxity in the skin (Schroeder et al., 2006; Morita, 2007; Vierkötter et al., 2010). Application of tobacco extracts to skin and oral fibroblasts *in vitro* triggered several hallmarks of senescence including premature cell cycle arrest, oxidative DNA damage, secretion of inflammatory cytokines and MMPs, and downregulation of cell junction proteins E-cadherin and ZO-1 (Coppé et al., 2008a). The ability to detect and quantify senescent cells in aged skin is indispensable to further elucidate how pollution may accelerate senescence and how this in turn alters skin appearance and function.

Whilst aging results in dry, itchy and more irritable skin, the underlying cause is likely multifactorial and involves changes in skin barrier function, androgen levels and sebaceous gland function (Kligman, 1979; Zouboulis and Boschnakow, 2001). Sebaceous glands contribute to skin oiliness and moisture by secreting a complex mix of lipids called sebum. While the number of sebaceous glands does not change during aging, its activity drastically decreases in postmenopausal females and males over 80 years old. Somewhat counterintuitive, contrasting its declining function and sebum production, the size of the sebaceous gland increases with age (Plewig and Kligman, 1978; Zouboulis and Boschnakow, 2001). Chronic UV exposure has also been implicated in sebaceous hyperplasia and carcinoma (Lesnik et al., 1992; Harwood et al., 2001). The specific involvement of cellular senescence in aging of the pigmentary units and sebaceous glands remains to be explored in greater detail.

## Genetic Diseases and Skin Aging

Multiple genetic syndromes that result in persistent DNA damage or telomere dysfunction (telomeropathies) and cellular senescence, predispose the skin to accelerated aging and/or carcinogenesis (Harada et al., 1999; Davis et al., 2007; Opresko and Shay, 2017). HGPS is a rare early-onset premature aging syndrome caused by a mutation in lamin A, resulting in a truncated mutant protein termed progerin. HGPS patients exhibit signs of accelerated aging at 12–18 months after birth and die in their mid-teens as a result of cardiovascular complications. Early clinical signs include alopecia, sclerotic skin, loss of subcutaneous fat and aberrant skin pigmentation (Merideth et al., 2008). At the cellular level, progerin expression results in atypical nuclear architecture, heterochromatin loss, DNA damage and premature senescence (Dreesen and Stewart, 2011; Vidak and Foisner, 2016; Kubben and Misteli, 2017). The primary driver mechanisms of these complex cellular phenotypes remain unclear. Progerin sequestration of NRF2, a major factor in a cell’s response to oxidative stress, has been suggested to play a role in progerin-dependent heterochromatin loss and DNA damage (Kubben et al., 2016). Other studies linked progerin-induced DNA damage to replication defects (Hilton et al., 2017; Wheaton et al., 2017) and telomere dysfunction (Kudlow et al., 2008; Decker et al., 2009; Benson et al., 2010; Chojnowski et al., 2015; Wood et al., 2015).

Mandibuloacral dysplasia (MAD) and restrictive dermopathy (RD) are also caused by nuclear lamina aberrations. Similar to HGPS, MAD is caused by a lamin A mutation and patients exhibit growth retardation, skeletal abnormalities, skin atrophy and hyperpigmentation (Novelli et al., 2002). RD is a rare neonatal lethal disease in which newborns exhibit intrauterine growth retardation, tight, erosive skin with hyperkeratosis, and sparse eyebrows and hair. RD is caused by loss of ZMPSTE24, a zinc metalloproteinase critical for the correct processing of lamin A (Navarro et al., 2005). While RD does not reflect skin aging phenotypes *per se*, the occurrence of these skin-specific laminopathies highlights the importance of nuclear lamina functions in the development and homeostasis of skin and hair.

Werner syndrome (WS), also referred to as adult-onset progeria, is regarded as a milder form of progeria. WS patients start to exhibit clinical features including hair loss and graying, scleroderma-like skin, ulcers and osteoporosis in their 30’s and die in their 50’s due to myocardial infarction and cancer. WS has been linked to mutations in *WRN*, a member of the RecQ helicase family (Yu et al., 1996) that lead to defects in DNA replication, repair, and premature cellular senescence (Oshima et al., 2017). Loss of function mutations in WRN result in defective telomere lagging strand synthesis, telomere loss and genomic instability (Crabbe et al., 2004, 2007).

Bloom syndrome is another disorder caused by loss of function mutations in the RecQ DNA helicase and affects DNA replication and repair (Bachrati and Hickson, 2003). Its clinical symptoms include the development of skin rash in sun-exposed areas, accelerated skin aging, alopecia, poikiloderma (irregular pigmentation, telangiectasia, and atrophy) and a predisposition to cancer (Goto, 2001; German and Sanz, 2013).

Xeroderma pigmentosum (XP) is a rare autosomal recessive syndrome characterized by extreme sensitivity to sunlight, resulting in sunburn, aberrant pigmentation, dry skin, premature skin aging, and increased risk to develop skin malignancies, including squamous cell carcinoma (SCC), basal cell carcinoma (BCC) and melanoma (Bukowska and Karwowski, 2018). The median age of death for XP patients is 29–32 years and most frequently caused by metastatic skin cancers and neurodegeneration (Bradford et al., 2011; DiGiovanna and Kraemer, 2012). The etiology of XP is characterized by defects in nucleotide excision repair (NER), a key pathway required to repair UVB-induced dipyrimidine photoproducts (Cleaver, 1968). Defects in NER lead to the accumulation of DNA damage, genomic instability, impaired cell growth, premature senescence and/or cell death (Niedernhofer et al., 2011; Black, 2016). Similarly, two other disorders caused by NER defects are Cockayne syndrome and trichothiodystrophy (TTD); both diseases are characterized by premature aging, neurological abnormalities and skin photosensitivity. Additionally, TTD patients display rough skin and brittle hair (Itin et al., 2001; Tan et al., 2005).

Mutations in ATM, an important molecule involved in activating checkpoint signaling in response to DNA double strand breaks, cause Ataxia-telangiectasia, another rare early-onset neurodegenerative disease in which patients display skin abnormalities including vitiligo, hair graying and telangiectasia in sun-exposed skin (Zou, 2007; Derheimer and Kastan, 2010; Rothblum-Oviatt et al., 2016).

Dyskeratosis congenita (DKC) is multi-systemic premature aging syndrome resulting in some skin defects. Initial clinical features appear during childhood and include abnormal skin pigmentation and nail dystrophy. During the second and third decade, patients develop bone marrow failure, ultimately resulting in their death. In addition, some patients exhibit an increased risk to develop premalignant and malignant lesions, including AK and SCC (Powell et al., 2014). DKC is caused by mutations in various components of telomerase (TCAP1, DKC1, TERC, TERT, NHP2, NOP10) or shelterin (TINF2), a protein complex involved in telomerase recruitment and telomere protection. These mutations result in impaired telomere maintenance, presumably in adult stem cell compartments, and premature telomere shortening (Mitchell et al., 1999; Batista et al., 2011; Dokal, 2011).

A common feature of the abovementioned genetic syndromes is the accumulation of persistent DNA damage, either by impaired DNA replication, repair, or signaling, as well as by DNA damage at unrepairable loci such as telomeres, resulting in premature cellular senescence and accelerated skin aging (Carrero et al., 2016). These phenotypes can be recapitulated in patient-derived fibroblasts, cell- or iPSC-based disease models and mouse models (Crabbe et al., 2004, 2007; Lombard et al., 2005; Batista et al., 2011; Zhang et al., 2011, 2015; Chojnowski et al., 2015). However, the rarity of these genetic diseases coupled with limited access to tissue biopsies have hampered efforts to test whether senescent cells accumulate within patient tissues *in vivo*. How are these accelerated aging syndromes relevant to normal aging? During chronological aging, at least the accumulation of genomic alterations correlates with the onset of age-related phenotypes and diseases (Chen et al., 2007; Vijg and Suh, 2013). Furthermore, an increase susceptibility to DNA damage (Xu et al., 2000) and decline in DNA repair capacity (Moriwaki et al., 1996; Yamada et al., 2006) have also been described in aging skin. Chronological aging can be accelerated by extrinsic factors that result in DNA damage, including UV radiation, particles from traffic pollution and cigarette smoke. In conclusion, increased DNA damage, impaired repair or signaling play a key conserved feature of premature aging syndromes and normal aging.

## Role of Cellular Senescence in Wounds and Cancers

Aging results in a progressive decline of skin function, leading to fragility, impaired barrier function, increased susceptibility to physical insults, infection, accumulation of age-related pathologies such as impaired wound healing and an increased risk of cancer (Lewis et al., 2011; Maru et al., 2014).

Skin wound healing occurs in four stages: hemostasis, inflammation, proliferation and remodeling. Wound closure takes place during the proliferation phase and depends on the formation of granulation tissue and the generation of contractile myofibroblasts. A ground breaking study by Demaria et al. (2014) demonstrated that senescent cells transiently accumulate during the proliferative stage and secrete platelet-derived growth factor AA (PDGF-AA) to induce myofibroblasts differentiation and maturation, essential for wound closure. Elimination of senescent cells reduced the number of myofibroblasts, delayed wound closure and increased fibrosis, all of which could be prevented by ectopic administration of PDGF-AA (Jun and Lau, 2010; Demaria et al., 2014). Similarly, senescent cells prevent fibrosis during liver regeneration (Krizhanovsky et al., 2008; Kim et al., 2013). These results demonstrate that senescent cells play an important role in tissue repair via cell-autonomous mechanisms: in the skin through the secretion of PDGF-AA; in the regenerating liver by secreting Mmp9, Mmp13 and IL-6. Thus, senescent cells may limit tissue regeneration in order to protect against unrestricted cell proliferation. On the flip side, a prolonged presence of senescent cells in the elderly prevents re-epithelialization and wound closure resulting in chronic wounds (Holt et al., 1992; Mendez et al., 1998; Vande Berg and Robson, 2003; Telgenhoff and Shroot, 2005; Vande Berg et al., 2005). Taken together, cellular senescence plays a complex role during normal wound healing as well as in chronic wounds.

## Senescence and Skin Cancer

Cellular senescence is a double-edged sword: on the one hand, the accumulation of senescent cells may contribute to various age-related complications; on the other hand, RS and OIS suppress uncontrolled proliferation of cells that are at risk for preneoplastic transformation, thereby limiting tumorigenesis. As a result, cells from premalignant clinical specimens of breast, colon, lung and bladder tumors exhibited persistent DNA damage checkpoint activation and expressed some senescence markers (Bartkova et al., 2005, 2006; Gorgoulis et al., 2005; Nuciforo et al., 2007; Suram et al., 2012). Besides promoting non-cell-autonomous growth inhibition, the production of SASP is also critical for the recruitment of immune cells that create an anti-tumorigenic microenvironment important for the clearance of senescent cells (Coppé et al., 2010).

In the skin, the vast majority of benign nevi are caused either by activating mutations in N-RAS or BRAF, and are unlikely to progress toward melanoma. Melanocytes within these preneoplastic lesions are positive for multiple senescence markers, including SA-β-gal, p16^INK4a^ and loss of lamin B1 (Michaloglou et al., 2005; Gray-Schopfer et al., 2006; Suram et al., 2012; Ivanov et al., 2013), which are absent in more advanced melanoma (Michaloglou et al., 2005; Suram et al., 2012). Kras mutant mice also expressed increased senescence markers in premalignant lung and pancreatic adenomas, and in chemically induced skin papillomas (Collado et al., 2005).

Skin cancer represents the most common human cancer type and its incidence increases with age and UV exposure (Narayanan et al., 2010). Long-term exposure to sunlight results in persistent DNA damage, increased cellular senescence and formation of premalignant lesions such as AK. AK affects approximately 60% of individuals above the age of 40 with a history of sun exposure. If left untreated, a percentage of AK lesions will progress toward invasive SCCs (Berman, 2012; Siegel et al., 2017). Clinically, 97% of SCCs are contiguous with AK (Hurwitz and Monger, 1995) yet the risk of progression or regression remains difficult to assess. Although cells within AK lesions exhibit genomic instability and telomere shortening (Rehman et al., 1994; Ikeda et al., 2014), it is unclear whether senescent cells accumulate within AK lesions. As senescent cells generally accumulate in premalignant, but not malignant lesions, the identification and quantification of senescent cells within AK lesions may serve as a valuable tool to diagnose and distinguish AK from SCC and predict the course of disease progression.

## Senescent Cells: Therapeutic Opportunities for Cancer and Aging

Numerous studies have aimed at preventing the accumulation of senescent cells within aging tissues. However, strategies that involve blocking p16^INK4a^ and p53, or activating telomerase to extend the proliferative capacity of cells inevitably lead to an increased cancer risk. Thus, a safer approach might be to either selectively eliminate senescent cells from tissues or modulate their function (particularly the SASP). The use of a progeroid mouse model expressing a mutated form of the mitotic checkpoint BubR1 has proven instrumental in demonstrating that p16^INK4a^-positive senescent cells drive age-related pathologies, and that selective elimination of these cells can prevent or delay age-related tissue deterioration (Baker et al., 2008, 2011). Similarly, clearance of p16^INK4a^-positive cells in normal-aging mice extended their median lifespan, delayed tumorigenesis, improved healthspan indices including heart and kidney function, and delayed age-related decline in exploratory behavior (Baker et al., 2016). Multiple recent studies confirmed that clearing senescent cells improved tissue function in various age-related pathologies including degenerative joint disease, hepatic fat accumulation and liver chemotoxicity (Xu et al., 2015; Baar et al., 2017; Farr et al., 2017; Jeon et al., 2017; Ogrodnik et al., 2017). These studies established senescent cells as potential therapeutic targets in the treatment of aging and age-related diseases. As a result, compounds that selectively eliminate senescent cells (senolytics) are under investigation to treat and prevent various age-related diseases (Baar et al., 2017; Kirkland and Tchkonia, 2017). While several molecular pathways that trigger senescence are under consideration for the development of senolytics, the majority are directed at the pathways that confer apoptosis-resistance in senescent cells (in particular the anti-apoptotic BCL protein family) (Chang et al., 2016; Yosef et al., 2016; Zhu et al., 2016). However, few studies have probed how treatment with senolytics may affect skin function and homeostasis. In one study involving a skin-specific keratin 5-driven *p14^ARF^* transgenic mouse model, senescent epidermal cells were efficiently eliminated upon delivery of ABT-737, a BCL-specific senolytic compound (Tokarsky-Amiel et al., 2013; Yosef et al., 2016). Although elimination of senescent cells from the epidermis led to increased proliferation within hair follicles, the underlying mechanism or other consequences on skin function were not investigated. Thus, it remains to be determined whether senolytics offer novel therapeutic approaches to treat chronic wounds, skin inflammation, age-related pigmentation disorders and ECM breakdown, or whether their clearance would impair wound healing or other skin functions in younger individuals.

A prerequisite for any such studies is a better understanding as to which skin compartment or cell type becomes senescent in the myriad age-related skin pathologies. Multiplexing senescence biomarkers with cell-type specific markers will pave the way to address this question. In addition, it is important to better understand how senescence affects or modulates the function of various skin cell types. To date, most SASP studies have focused on dermal fibroblasts and little is known about the SASP of other skin resident cell types, including melanocytes, keratinocytes or cell types from skin appendages, such as sebocytes. This is particularly relevant in skin as the interplay between these different cell types is essential to regulate skin functions including pigmentation, barrier function, sebum production, wound healing and cancer progression.

## Conclusion

The recognition of the role of cellular senescence in tissue aging has progressed tremendously in the past decade and this is in part attributed to better characterization of senescence biomarkers which allows qualitative and quantitative detection of senescent cells *in vitro* and *in situ*. Recent studies provided evidence that senescent cells accumulate in aged skin and may be driving the functional deterioration that characterizes aging skin and age-related skin diseases. Improved methodologies to detect senescent cells and a better understanding how senescence affects cellular function will enable us to determine how they contribute to various age-related skin changes and pathologies, including impaired wound healing and tumorigenesis. Moreover, the careful evaluation of senolytics in animal models and 3D reconstructed skin organotypics will facilitate the successful translation of senolytics as a therapeutic intervention for skin aging. The accessibility of human skin offers advantages to test these hypotheses.

## Author Contributions

OD and AW wrote the manuscript and prepared the figures.

## Conflict of Interest Statement

The authors declare that the research was conducted in the absence of any commercial or financial relationships that could be construed as a potential conflict of interest.

## References

[B1] Acosta J. C. O’Loghlen A. Banito A. Guijarro M. V. Augert A. Raguz S. (2008). Chemokine signaling via the CXCR2 receptor reinforces senescence. *Cell* 133 1006–1018. 10.1016/j.cell.2008.03.038 18555777

[B2] Allsopp R. C. Vaziri H. Patterson C. Goldstein S. Younglai E. V. Futcher A. B. (1992). Telomere length predicts replicative capacity of human fibroblasts. *Proc. Natl. Acad. Sci. U.S.A.* 89 10114–10118. 10.1073/pnas.89.21.10114 1438199 PMC50288

[B3] Baar M. P. Brandt R. M. C. Putavet D. A. Klein J. D. D. Derks K. W. J. Bourgeois B. R. M. (2017). Targeted apoptosis of senescent cells restores tissue homeostasis in response to chemotoxicity and aging. *Cell* 169 132–147.e16. 10.1016/j.cell.2017.02.031 28340339 PMC5556182

[B4] Bachrati C. Z. Hickson I. D. (2003). RecQ helicases: suppressors of tumorigenesis and premature aging. *Biochem. J.* 374 577–606. 10.1042/bj20030491 12803543 PMC1223634

[B5] Baker D. J. Childs B. G. Durik M. Wijers M. E. Sieben C. J. Zhong J. (2016). Naturally occurring p16 Ink4a-positive cells shorten healthy lifespan. *Nature* 530 184–189. 10.1038/nature16932 26840489 PMC4845101

[B6] Baker D. J. Perez-Terzic C. Jin F. Pitel K. Niederländer N. J. Jeganathan K. (2008). Opposing roles for p16Ink4a and p19Arf in senescence and ageing caused by BubR1 insufficiency. *Nat. Cell Biol.* 10 825–836. 10.1038/ncb1744 18516091 PMC2594014

[B7] Baker D. J. Wijshake T. Tchkonia T. Lebrasseur N. K. Childs B. G. Van De Sluis B. (2011). Clearance of p16 Ink4a-positive senescent cells delays ageing-associated disorders. *Nature* 479 232–236. 10.1038/nature10600 22048312 PMC3468323

[B8] Bakkenist C. J. Kastan M. B. (2003). DNA damage activates ATM through intermolecular autophosphorylation and dimer dissociation. *Nature* 421 499–506. 10.1038/nature01368 12556884

[B9] Bartkova J. Hořejší Z. Koed K. Krämer A. Tort F. Zleger K. (2005). DNA damage response as a candidate anti-cancer barrier in early human tumorigenesis. *Nature* 434 864–870. 10.1038/nature03482 15829956

[B10] Bartkova J. Rezaei N. Liontos M. Karakaidos P. Kletsas D. Issaeva N. (2006). Oncogene-induced senescence is part of the tumorigenesis barrier imposed by DNA damage checkpoints. *Nature* 444 633–637. 10.1038/nature05268 17136093

[B11] Batista L. F. Pech M. F. Zhong F. L. Nguyen H. N. Xie K. T. Zaug A. J. (2011). Telomere shortening and loss of self-renewal in dyskeratosis congenita induced pluripotent stem cells. *Nature* 474 399–402. 10.1038/nature10084 21602826 PMC3155806

[B12] Behrens A. Van Deursen J. M. Rudolph K. L. Schumacher B. (2014). Impact of genomic damage and ageing on stem cell function. *Nat. Cell Biol.* 16 201–207. 10.1038/ncb2928 24576896 PMC4214082

[B13] Benson E. K. Lee S. W. Aaronson S. A. (2010). Role of progerin-induced telomere dysfunction in HGPS premature cellular senescence. *J. Cell Sci.* 123 2605–2612. 10.1242/jcs.067306 20605919 PMC2908049

[B14] Berman B. (2012). New developments in the treatment of actinic keratosis: focus on ingenol mebutate gel. *Clin. Cosmet. Investig. Dermatol.* 5 111–122. 10.2147/CCID.S28905 22956883 PMC3430094

[B15] Berneburg M. Gattermann N. Stege H. Grewe M. Vogelsang K. Ruzicka T. (1997). Chronically ultraviolet-exposed human skin shows a higher mutation frequency of mitochondrial DNA as compared to unexposed skin and the hematopoietic system. *Photochem. Photobiol.* 66 271–275. 10.1111/j.1751-1097.1997.tb08654.x 9277148

[B16] Bianchi M. E. (2006). DAMPs, PAMPs and alarmins: all we need to know about danger. *J. Leukoc. Biol.* 81 1–5. 10.1189/jlb.0306164 17032697

[B17] Biran A. Zada L. Abou Karam P. Vadai E. Roitman L. Ovadya Y. (2017). Quantitative identification of senescent cells in aging and disease. *Aging Cell* 16 661–671. 10.1111/acel.12592 28455874 PMC5506427

[B18] Black J. O. (2016). Xeroderma pigmentosum. *Head Neck Pathol.* 10 139–144. 10.1007/s12105-016-0707-8 26975629 PMC4838978

[B19] Blackburn E. H. Challoner P. B. (1984). Identification of a telomeric DNA sequence in *Trypanosoma brucei*. *Cell* 36 447–457. 10.1016/0092-8674(84)90238-1 6319025

[B20] Blanpain C. Fuchs E. (2006). Epidermal stem cells of the skin. *Annu. Rev. Cell Dev. Biol.* 22 339–373. 10.1146/annurev.cellbio.22.010305.104357 16824012 PMC2405915

[B21] Bradford P. T. Goldstein A. M. Tamura D. Khan S. G. Ueda T. Boyle J. (2011). Cancer and neurologic degeneration in xeroderma pigmentosum: long term follow-up characterises the role of DNA repair. *J. Med. Genet.* 48 168–176. 10.1136/jmg.2010.083022 21097776 PMC3235003

[B22] Bukowska B. Karwowski B. T. (2018). Actual state of knowledge in the field of diseases related with defective nucleotide excision repair. *Life Sci.* 195 6–18. 10.1016/j.lfs.2017.12.035 29305302

[B23] Campisi J. d’Adda di Fagagna F. (2007). Cellular senescence: when bad things happen to good cells. *Nat. Rev. Mol. Cell Biol.* 8 729–740. 10.1038/nrm2233 17667954

[B24] Carrero D. Soria-Valles C. López-Otín C. (2016). Hallmarks of progeroid syndromes: lessons from mice and reprogrammed cells. *Dis. Model. Mech.* 9 719–735. 10.1242/dmm.024711 27482812 PMC4958309

[B25] Chainiaux F. Magalhaes J.-P. Eliaers F. Remacle J. Toussaint O. (2002). UVB-induced premature senescence of human diploid skin fibroblasts. *Int. J. Biochem. Cell Biol.* 34 1331–1339. 10.1016/S1357-2725(02)00022-5 12200029

[B26] Chang J. Wang Y. Shao L. Laberge R. M. Demaria M. Campisi J. (2016). Clearance of senescent cells by ABT263 rejuvenates aged hematopoietic stem cells in mice. *Nat. Med.* 22 78–83. 10.1038/nm.4010 26657143 PMC4762215

[B27] Chen J. H. Hales C. N. Ozanne S. E. (2007). DNA damage, cellular senescence and organismal ageing: causal or correlative? *Nucleic Acids Res.* 35 7417–7428. 10.1093/nar/gkm681 17913751 PMC2190714

[B28] Chen Q. M. Tu V. C. Catania J. Burton M. Toussaint O. Dilley T. (2000). Involvement of Rb family proteins, focal adhesion proteins and protein synthesis in senescent morphogenesis induced by hydrogen peroxide. *J. Cell Sci.* 113 4087–4097. 11058095 10.1242/jcs.113.22.4087

[B29] Chen W. Kang J. Xia J. Li Y. Yang B. Chen B. (2008). p53-related apoptosis resistance and tumor suppression activity in UVB-induced premature senescent human skin fibroblasts. *Int. J. Mol. Med.* 21 645–653. 10.3892/ijmm.21.5.645 18425358

[B30] Chojnowski A. Ong P. F. Wong E. S. M. Lim J. S. Y. Mutalif R. A. Navasankari R. (2015). Progerin reduces LAP2a-telomere association in Hutchinson-Gilford progeria. *eLife* 4 1–21. 10.7554/eLife.07759 26312502 PMC4565980

[B31] Chung J. H. (2003). Photoaging in Asians. *Photodermatol. Photoimmunol. Photomed.* 19 109–121. 10.1034/j.1600-0781.2003.00027.x 12914595

[B32] Cleaver J. E. (1968). Defective repair replication of DNA in xeroderma pigmentosum. *Nature* 218 652–656. 10.1038/218652a0 5655953

[B33] Collado M. Gil J. Efeyan A. Guerra C. Schuhmacher A. J. Barradas M. (2005). Tumour biology: senescence in premalignant tumours. *Nature* 436:642. 10.1038/436642a 16079833

[B34] Collado M. Serrano M. (2010). Senescence in tumours: evidence from mice and humans. *Nat. Rev. Cancer* 10 51–57. 10.1038/nrc2772 20029423 PMC3672965

[B35] Contrepois K. Coudereau C. Benayoun B. A. Schuler N. Roux P. F. Bischof O. (2017). Histone variant H2A.J accumulates in senescent cells and promotes inflammatory gene expression. *Nat. Commun.* 8:14995. 10.1038/ncomms14995 28489069 PMC5436145

[B36] Coppé J.-P. Boysen M. Sun C. H. Wong B. J. F. Kang M. K. Park N.-H. (2008a). A role for fibroblasts in mediating the effects of tobacco-induced epithelial cell growth and invasion. *Mol. Cancer Res.* 6 1085–1098. 10.1158/1541-7786.MCR-08-0062 18644973 PMC2768668

[B37] Coppé J.-P. Patil C. K. Rodier F. Sun Y. Muñoz D. P. Goldstein J. (2008b). Senescence-associated secretory phenotypes reveal cell-nonautonomous functions of oncogenic RAS and the p53 tumor suppressor. *PLoS Biol.* 6:e301. 10.1371/journal.pbio.0060301 19053174 PMC2592359

[B38] Coppé J.-P. Desprez P.-Y. Krtolica A. Campisi J. (2010). The senescence-associated secretory phenotype: the dark side of tumor suppression. *Annu. Rev. Pathol. Mech. Dis.* 5 99–118. 10.1146/annurev-pathol-121808-102144 20078217 PMC4166495

[B39] Coppé J. P. Rodier F. Patil C. K. Freund A. Desprez P. Y. Campisi J. (2011). Tumor suppressor and aging biomarker p16(INK4a) induces cellular senescence without the associated inflammatory secretory phenotype. *J. Biol. Chem.* 286 36396–36403. 10.1074/jbc.M111.257071 21880712 PMC3196093

[B40] Crabbe L. Jauch A. Naeger C. M. Holtgreve-Grez H. Karlseder J. (2007). Telomere dysfunction as a cause of genomic instability in Werner syndrome. *Proc. Natl. Acad. Sci. U.S.A.* 104 2205–2210. 10.1073/pnas.0609410104 17284601 PMC1794219

[B41] Crabbe L. Verdun R. E. Haggblom C. I. Karlseder J. (2004). Defective telomere lagging strand synthesis in cells lacking WRN helicase activity. *Science* 306 1951–1953. 10.1126/science.1103619 15591207

[B42] D’Adda Di Fagagna F. Reaper P. M. Clay-Farrace L. Fiegler H. Carr P. Von Zglinicki T. (2003). A DNA damage checkpoint response in telomere-initiated senescence. *Nature* 426 194–198. 10.1038/nature02118 14608368

[B43] Davalos A. R. Kawahara M. Malhotra G. K. Schaum N. Huang J. Ved U. (2013). p53-dependent release of alarmin HMGB1 is a central mediator of senescent phenotypes. *J. Cell Biol.* 201 613–629. 10.1083/jcb.201206006 23649808 PMC3653366

[B44] Davis T. Wyllie F. S. Rokicki M. J. Bagley M. C. Kipling D. (2007). The role of cellular senescence in Werner syndrome: toward therapeutic intervention in human premature aging. *Ann. N. Y. Acad. Sci.* 1100 455–469. 10.1196/annals.1395.051 17460211

[B45] de Gruijl F. R. (1999). Skin cancer and solar UV radiation. *Eur. J. Cancer* 35 2003–2009. 10.1016/S0959-8049(99)00283-X 10711242

[B46] De Snoo F. A. Bishop D. T. Bergman W. Van Leeuwen I. Van Der Drift C. Van Nieuwpoort F. A. (2008). Increased risk of cancer other than melanoma in CDKN2A founder mutation (p16-Leiden)-positive melanoma families. *Clin. Cancer Res.* 14 7151–7157. 10.1158/1078-0432.CCR-08-0403 18981015

[B47] Debacq-Chainiaux F. Borlon C. Pascal T. Royer V. Eliaers F. Ninane N. (2005). Repeated exposure of human skin fibroblasts to UVB at subcytotoxic level triggers premature senescence through the TGF-beta1 signaling pathway. *J. Cell Sci.* 118 743–758. 10.1242/jcs.01651 15671065

[B48] Debacq-Chainiaux F. Erusalimsky J. D. Campisi J. Toussaint O. (2009). Protocols to detect senescence-associated beta-galactosidase (SA-betagal) activity, a biomarker of senescent cells in culture and in vivo. *Nat. Protoc.* 4 1798–1806. 10.1038/nprot.2009.191 20010931

[B49] Decker M. L. Chavez E. Vulto I. Lansdorp P. M. (2009). Telomere length in hutchinson-gilford progeria syndrome. *Mech. Ageing Dev.* 130 377–383. 10.1016/j.mad.2009.03.001 19428457

[B50] Demaria M. Ohtani N. Youssef S. A. Rodier F. Toussaint W. Mitchell J. R. (2014). An essential role for senescent cells in optimal wound healing through secretion of PDGF-AA. *Dev. Cell* 31 722–733. 10.1016/j.devcel.2014.11.012 25499914 PMC4349629

[B51] Derheimer F. A. Kastan M. B. (2010). Multiple roles of ATM in monitoring and maintaining DNA integrity. *FEBS Lett.* 584 3675–3681. 10.1016/j.febslet.2010.05.031 20580718 PMC2950315

[B52] DiGiovanna J. J. Kraemer K. H. (2012). Shining a light on xeroderma pigmentosum. *J. Invest. Dermatol.* 132 785–796. 10.1038/jid.2011.426 22217736 PMC3279615

[B53] Dimri G. P. Lee X. Basile G. Acosta M. Scott G. Roskelley C. (1995). A biomarker that identifies senescent human cells in culture and in aging skin in vivo. *Proc. Natl. Acad. Sci. U.S.A.* 92 9363–9367. 10.1073/pnas.92.20.9363 7568133 PMC40985

[B54] Dokal I. (2011). Dyskeratosis congenita. *Hematol. Am. Soc. Hematol. Educ. Progr.* 2011 480–486. 10.1182/asheducation-2011.1.480 22160078

[B55] Dou Z. Xu C. Donahue G. Shimi T. Pan J.-A. Zhu J. (2015). Autophagy mediates degradation of nuclear lamina. *Nature* 527 105–109. 10.1038/nature15548 26524528 PMC4824414

[B56] Dreesen O. Chojnowski A. Ong P. F. Zhao T. Y. Common J. E. Lunny D. (2013a). Lamin B1 fluctuations have differential effects on cellular proliferation and senescence. *J. Cell Biol.* 200 605–617. 10.1083/jcb.201206121 23439683 PMC3587829

[B57] Dreesen O. Ong P. F. Chojnowski A. Colman A. (2013b). The contrasting roles of lamin B1 in cellular aging and human disease. *Nucleus* 4 283–290. 10.4161/nucl.25808 23873483 PMC3810336

[B58] Dreesen O. Stewart C. L. (2011). Accelerated aging syndromes, are they relevant to normal human aging? *Aging* 3 889–895. 10.18632/aging.100383 21931180 PMC3227453

[B59] Duval C. Cohen C. Chagnoleau C. Flouret V. Bourreau E. Bernerd F. (2014). Key regulatory role of dermal fibroblasts in pigmentation as demonstrated using a reconstructed skin model: impact of photo-aging. *PLoS One* 9:e114182. 10.1371/journal.pone.0114182 25490395 PMC4260844

[B60] Farr J. N. Xu M. Weivoda M. M. Monroe D. G. Fraser D. G. Onken J. L. (2017). Targeting cellular senescence prevents age-related bone loss in mice. *Nat. Med.* 23 1072–1079. 10.1038/nm.4385 28825716 PMC5657592

[B61] Freund A. Laberge R.-M. Demaria M. Campisi J. (2012). Lamin B1 loss is a senescence-associated biomarker. *Mol. Biol. Cell* 23 2066–2075. 10.1091/mbc.E11-10-0884 22496421 PMC3364172

[B62] German J. Sanz M. (eds). (2013). “Bloom’s syndrome,” in *Brenner’s Encyclopedia of Genetics*, (New York, NY: Elsevier), 353–355. 10.1016/B978-0-12-374984-0.00160-1

[B63] Ghosh K. Capell B. C. (2016). The senescence-associated secretory phenotype: critical effector in skin cancer and aging. *J. Invest. Dermatol.* 136 2133–2139. 10.1016/j.jid.2016.06.621 27543988 PMC5526201

[B64] Gilchrest B. A. Blog F. B. Szabo G. (1979). Effects of aging and chronic sun exposure on melanocytes in human skin. *J. Invest. Dermatol.* 73 141–143. 10.1111/1523-1747.ep12581580 88488

[B65] Going J. J. Stuart R. C. Downie M. Fletcher-Monaghan A. J. Keith W. N. (2002). “Senescence-associated” beta-galactosidase activity in the upper gastrointestinal tract. *J. Pathol.* 196 394–400. 10.1002/path.1059 11920734

[B66] Goldstein S. Moerman E. J. Fujii S. Sobel B. E. (1994). Overexpression of plasminogen activator inhibitor type-1 in senescent fibroblasts from normal subjects and those with Werner syndrome. *J. Cell. Physiol.* 161 571–579. 10.1002/jcp.1041610321 7962138

[B67] Gorgoulis V. G. Vassiliou L.-V. F. Karakaidos P. Zacharatos P. Kotsinas A. Liloglou T. (2005). Activation of the DNA damage checkpoint and genomic instability in human precancerous lesions. *Nature* 434 907–913. 10.1038/nature03485 15829965

[B68] Goto M. (2001). Clinical characteristics of Werner syndrome and other premature aging syndromes: pattern of aging in progeroid syndromes. *Gann Monogr. Cancer Res.* 49 27–39.

[B69] Gray-Schopfer V. C. Cheong S. C. Chong H. Chow J. Moss T. Abdel-Malek Z. A. (2006). Cellular senescence in naevi and immortalisation in melanoma: a role for p16? *Br. J. Cancer* 95 496–505. 10.1038/sj.bjc.6603283 16880792 PMC2360676

[B70] Grönniger E. Weber B. Heil O. Peters N. Stäb F. Wenck H. (2010). Aging and chronic sun exposure cause distinct epigenetic changes in human skin. *PLoS Genet.* 6:e1000971. 10.1371/journal.pgen.1000971 20523906 PMC2877750

[B71] Harada Y. N. Shiomi N. Koike M. Ikawa M. Okabe M. Hirota S. (1999). Postnatal growth failure, short life span, and early onset of cellular senescence and subsequent immortalization in mice lacking the xeroderma pigmentosum group G gene. *Mol. Cell. Biol.* 19 2366–2372. 10.1128/MCB.19.3.2366 10022922 PMC84028

[B72] Harwood C. A. Swale V. J. Bataille V. A. Quinn A. G. Ghali L. Patel S. V. (2001). An association between sebaceous carcinoma and microsatellite instability in immunosuppressed organ transplant recipients. *J. Invest. Dermatol.* 116 246–253. 10.1046/j.1523-1747.2001.01233.x 11180000

[B73] Hayflick L. (1965). The limited in vitro lifetime of human diploid cell strains. *Exp. Cell Res.* 37 614–636. 10.1016/0014-4827(65)90211-9 14315085

[B74] Hayflick L. Moorhead P. S. (1961). The serial cultivation of human diploid cell strains. *Exp. Cell Res.* 25 585–621. 10.1016/0014-4827(61)90192-6 13905658

[B75] He S. Sharpless N. E. (2017). Senescence in health and disease. *Cell* 169 1000–1011. 10.1016/j.cell.2017.05.015 28575665 PMC5643029

[B76] Herbig U. Ferreira M. Condel L. Carey D. Sedivy J. M. (2006). Cellular senescence in aging primates. *Science* 311:1257. 10.1126/science.1122446 16456035

[B77] Hewitt G. Jurk D. Marques F. D. M. Correia-Melo C. Hardy T. Gackowska A. (2012). Telomeres are favoured targets of a persistent DNA damage response in ageing and stress-induced senescence. *Nat. Commun.* 3:708. 10.1038/ncomms1708 22426229 PMC3292717

[B78] Hilton B. A. Liu J. Cartwright B. M. Liu Y. Breitman M. Wang Y. (2017). Progerin sequestration of PCNA promotes replication fork collapse and mislocalization of XPA in laminopathy-related progeroid syndromes. *FASEB J.* 31 3882–3893. 10.1096/fj.201700014R 28515154 PMC5572696

[B79] Hoger T. H. Krohne G. Franke W. W. (1988). Amino acid sequence and molecular characterization of murine lamin B as deduced from cDNA clones. *Eur. J. Cell Biol.* 47 283–290. 3243285

[B80] Holt D. R. Kirk S. J. Regan M. C. Hurson M. Lindblad W. J. Barbul A. (1992). Effect of age on wound healing in healthy human beings. *Surgery* 112 293–297; discussion 297–298. 10.5555/URI:PII:003960609290223M 1641768

[B81] Horvath S. (2013). DNA methylation age of human tissues and cell types. *Genome Biol.* 14:3156. 10.1186/gb-2013-14-10-r115 24138928 PMC4015143

[B82] Hüls A. Vierkötter A. Gao W. Krämer U. Yang Y. Ding A. (2016). Traffic-related air pollution contributes to development of facial lentigines: further epidemiological evidence from Caucasians and Asians. *J. Invest. Dermatol.* 136 1053–1056. 10.1016/j.jid.2015.12.045 26868871

[B83] Hurwitz R. M. Monger L. E. (1995). Solar Keratosis: an evolving squamous cell carcinoma. benign or malignant? *Dermatol. Surg.* 21 184–184. 10.1111/j.1524-4725.1995.tb00141.x 7894943

[B84] Ichihashi M. Ueda M. Budiyanto A. Bito T. Oka M. Fukunaga M. (2003). UV-induced skin damage. *Toxicology* 189 21–39. 10.1016/S0300-483X(03)00150-1 12821280

[B85] Ikeda H. Aida J. Hatamochi A. Hamasaki Y. Izumiyama-Shimomura N. Nakamura K. I. (2014). Quantitative fluorescence in situ hybridization measurement of telomere length in skin with/without sun exposure or actinic keratosis. *Hum. Pathol.* 45 473–480. 10.1016/j.humpath.2013.10.009 24411948

[B86] Itin P. H. Sarasin A. Pittelkow M. R. (2001). Trichothiodystrophy: update on the sulfur deficient brittle hair syndromes. *J. Am. Acad. Dermatol.* 44 891–920. 10.1067/mjd.2001.114294 11369901

[B87] Ivanov A. Pawlikowski J. Manoharan I. Tuyn J. Van Nelson D. M. Singh Rai T. (2013). Lysosome-mediated processing of chromatin in senescence. *J. Cell Biol.* 202 129–143. 10.1083/jcb.201212110 23816621 PMC3704985

[B88] Jeon O. H. Kim C. Laberge R. M. Demaria M. Rathod S. Vasserot A. P. (2017). Local clearance of senescent cells attenuates the development of post-traumatic osteoarthritis and creates a pro-regenerative environment. *Nat. Med.* 23 775–781. 10.1038/nm.4324 28436958 PMC5785239

[B89] Jeyapalan J. C. Ferreira M. Sedivy J. M. Herbig U. (2007). Accumulation of senescent cells in mitotic tissue of aging primates. *Mech. Ageing Dev.* 128 36–44. 10.1016/j.mad.2006.11.008 17116315 PMC3654105

[B90] Johnson K. E. Wulff B. C. Oberyszyn T. M. Wilgus T. A. (2013). Ultraviolet light exposure stimulates HMGB1 release by keratinocytes. *Arch. Dermatol. Res.* 305 805–815. 10.1007/s00403-013-1401-2 23942756 PMC3818797

[B91] Jun J. Lau L. F. (2010). The matricellular protein CCN1 induces fibroblast senescence and restricts fibrosis in cutaneous wound healing. *Nat. Cell Biol.* 12 676–685. 10.1038/ncb2070 20526329 PMC2919364

[B92] Kim K.-H. Chen C.-C. Monzon R. I. Lau L. F. (2013). Matricellular protein CCN1 promotes regression of liver fibrosis through induction of cellular senescence in hepatic myofibroblasts. *Mol. Cell. Biol.* 33 2078–2090. 10.1128/MCB.00049-13 23508104 PMC3647960

[B93] Kirkland J. L. Tchkonia T. (2017). Cellular senescence: a translational perspective. *eBiomedicine* 21 21–28. 10.1016/j.ebiom.2017.04.013 28416161 PMC5514381

[B94] Kligman A. M. (1979). Perspectives and problems in cutaneous gerontology. *J. Invest. Dermatol.* 73 39–46. 10.1111/1523-1747.ep12532758 156237

[B95] Koch H. Wittern K. P. Bergemann J. (2001). In human keratinocytes the common deletion reflects donor variabilities rather than chronologic aging and can be induced by ultraviolet A irradiation. *J. Invest. Dermatol.* 117 892–897. 10.1046/j.0022-202X.2001.01513.x 11676829

[B96] Kortlever R. M. Higgins P. J. Bernards R. (2006). Plasminogen activator inhibitor-1 is a critical downstream target of p53 in the induction of replicative senescence. *Nat. Cell Biol.* 8 877–884. 10.1038/ncb1448 16862142 PMC2954492

[B97] Kosar M. Bartkova J. Hubackova S. Hodny Z. Lukas J. Bartek J. (2011). Senescence-associated heterochromatin foci are dispensable for cellular senescence, occur in a cell type- and insult-dependent manner, and follow expression of p16ink4a. *Cell Cycle* 10 457–468. 10.4161/cc.10.3.14707 21248468

[B98] Krishnamurthy J. Torrice C. Ramsey M. R. Kovalev G. I. Al-Regaiey K. Su L. (2004). Ink4a/Arf expression is a biomarker of aging. *J. Clin. Invest.* 114 1299–1307. 10.1172/JCI200422475 15520862 PMC524230

[B99] Krizhanovsky V. Yon M. Dickins R. A. Hearn S. Simon J. Miething C. (2008). Senescence of activated stellate cells limits liver fibrosis. *Cell* 134 657–667. 10.1016/j.cell.2008.06.049 18724938 PMC3073300

[B100] Kubben N. Misteli T. (2017). Shared molecular and cellular mechanisms of premature ageing and ageing-associated diseases. *Nat. Rev. Mol. Cell Biol.* 18 595–609. 10.1038/nrm.2017.68 28792007 PMC6290461

[B101] Kubben N. Zhang W. Wang L. Voss T. C. Yang J. Qu J. (2016). Repression of the antioxidant NRF2 pathway in premature aging. *Cell* 165 1361–1374. 10.1016/j.cell.2016.05.017 27259148 PMC4893198

[B102] Kudlow B. A. Stanfel M. N. Burtner C. R. Johnston E. D. Kennedy B. K. (2008). Suppression of Proliferative defects associated with processing-defective lamin A mutants by hTERT or inactivation of p53. *Mol. Biol. Cell* 19 5238–5248. 10.1091/mbc.E08-05-0492 18843043 PMC2592682

[B103] Kuilman T. Michaloglou C. Mooi W. J. Peeper D. S. (2010). The essence of senescence. *Genes Dev.* 24 2463–2479. 10.1101/gad.1971610 21078816 PMC2975923

[B104] Kuilman T. Michaloglou C. Vredeveld L. C. W. Douma S. van Doorn R. Desmet C. J. (2008). Oncogene-induced senescence relayed by an interleukin-dependent inflammatory network. *Cell* 133 1019–1031. 10.1016/j.cell.2008.03.039 18555778

[B105] Kuilman T. Peeper D. S. (2009). Senescence-messaging secretome: SMS-ing cellular stress. *Nat. Rev. Cancer* 9 81–94. 10.1038/nrc2560 19132009

[B106] Lavker R. M. Zheng P. S. Dong G. (1986). Morphology of aged skin. *Dermatol. Clin.* 4 379–389. 3521984

[B107] Lee B. Y. Han J. A. Im J. S. Morrone A. Johung K. Goodwin E. C. (2006). Senescence-associated beta-galactosidase is lysosomal beta-galactosidase. *Aging Cell* 5 187–195. 10.1111/j.1474-9726.2006.00199.x 16626397

[B108] Lesnik R. H. Kligman L. H. Kligman A. M. (1992). Agents that cause enlargement of sebaceous glands in hairless mice. I. Topical substances. *Arch. Dermatol. Res.* 284 100–105. 10.1007/BF00373378 1610208

[B109] Lewis D. A. Travers J. B. Machado C. Somani A. K. Spandau D. F. (2011). Reversing the aging stromal phenotype prevents carcinoma initiation. *Aging* 3 407–416. 21515933 10.18632/aging.100318 PMC3117456

[B110] Lewis D. A. Yi Q. Travers J. B. Spandau D. F. (2008). UVB-induced senescence in human keratinocytes requires a functional insulin-like growth factor-1 receptor and p53. *Mol. Biol. Cell* 19 1346–1353. 10.1091/mbc.E07-10-1041 18216278 PMC2291419

[B111] Lin S.-T. Fu Y.-H. (2009). miR-23 regulation of lamin B1 is crucial for oligodendrocyte development and myelination. *Dis. Model. Mech.* 2 178–188. 10.1242/dmm.001065 19259393 PMC2650193

[B112] Lombard D. B. Chua K. F. Mostoslavsky R. Franco S. Gostissa M. Alt F. W. (2005). DNA repair, genome stability, and aging. *Cell* 120 497–512. 10.1016/j.cell.2005.01.028 15734682

[B113] López-Otín C. Blasco M. A. Partridge L. Serrano M. Kroemer G. (2013). The hallmarks of aging. *Cell* 153 1194–1217. 10.1016/j.cell.2013.05.039 23746838 PMC3836174

[B114] Lukášová E. Kovařík A. Bačíková A. Falk M. Kozubek S. (2017). Loss of lamin B receptor is necessary to induce cellular senescence. *Biochem. J.* 474 281–300. 10.1042/BCJ20160459 27760841

[B115] Ma W. Wlaschek M. Tantcheva-Poór I. Schneider L. A. Naderi L. Razi-Wolf Z. (2001). Chronological ageing and photoageing of the fibroblasts and the dermal connective tissue. *Clin. Exp. Dermatol.* 26 592–599. 10.1046/j.1365-2230.2001.00905.x 11696063

[B116] Maru G. B. Gandhi K. Ramchandani A. Kumar G. (2014). The role of inflammation in skin cancer. *Adv. Exp. Med. Biol.* 816 437–469. 10.1007/978-3-0348-0837-8_17 24818733

[B117] McCart E. A. Thangapazham R. L. Lombardini E. D. Mog S. R. Panganiban R. A. M. Dickson K. M. (2017). Accelerated senescence in skin in a murine model of radiation-induced multi-organ injury. *J. Radiat. Res.* 58 636–646. 10.1093/jrr/rrx008 28340212 PMC5737212

[B118] Mendez M. V. Stanley A. Park H. Y. Shon K. Phillips T. Menzoian J. O. (1998). Fibroblasts cultured from venous ulcers display cellular characteristics of senescence. *J. Vasc. Surg.* 28 876–883. 10.1016/S0741-5214(98)70064-3 9808856

[B119] Merideth M. A. Gordon L. B. Clauss S. Sachdev V. Smith A. C. M. Perry M. B. (2008). Phenotype and course of hutchinson–gilford progeria syndrome. *N. Engl. J. Med.* 358 592–604. 10.1056/NEJMoa0706898 18256394 PMC2940940

[B120] Michaloglou C. Vredeveld L. C. W. Soengas M. S. Denoyelle C. Kuilman T. van der Horst C. M. A. M. (2005). BRAFE600-associated senescence-like cell cycle arrest of human naevi. *Nature* 436 720–724. 10.1038/nature03890 16079850

[B121] Mimeault M. Batra S. K. (2010). Recent advances on skin-resident stem/progenitor cell functions in skin regeneration, aging and cancers and novel anti-aging and cancer therapies. *J. Cell. Mol. Med.* 14 116–134. 10.1111/j.1582-4934.2009.00885.x 19725922 PMC2916233

[B122] Misteli T. Scaffidi P. (2005). Genome instability in progeria: when repair gets old. *Nat. Med.* 11 718–719. 10.1038/nm0705-718 16015360

[B123] Mitchell J. R. Wood E. Collins K. (1999). A telomerase component is defective in the human disease dyskeratosis congenita. *Nature* 402 551–555. 10.1038/990141 10591218

[B124] Montagna W. Carlisle K. (1979). Structural changes in aging human skin. *J. Invest. Dermatol.* 73 47–53. 10.1111/1523-1747.ep12532761 448177

[B125] Morita A. (2007). Tobacco smoke causes premature skin aging. *J. Dermatol. Sci.* 48 169–175. 10.1016/j.jdermsci.2007.06.015 17951030

[B126] Moriwaki S. I. Ray S. Tarone R. E. Kraemer K. H. Grossman L. (1996). The effect of donor age on the processing of UV-damaged DNA by cultured human cells: reduced DNA repair capacity and increased DNA mutability. *Mutat. Res.* 364 117–123. 10.1016/0921-8777(96)00029-8 8879277

[B127] Munro J. Barr N. I. Ireland H. Morrison V. Parkinson E. K. (2004). Histone deacetylase inhibitors induce a senescence-like state in human cells by a p16-dependent mechanism that is independent of a mitotic clock. *Exp. Cell Res.* 295 525–538. 10.1016/j.yexcr.2004.01.017 15093749

[B128] Murano S. Thweatt R. Shmookler Reis R. J. Jones R. A. Moerman E. J. Goldstein S. (1991). Diverse gene sequences are overexpressed in werner syndrome fibroblasts undergoing premature replicative senescence. *Mol. Cell. Biol.* 11 3905–3914. 10.1128/MCB.11.8.3905.Updated 1712899 PMC361182

[B129] Narayanan D. L. Saladi R. N. Fox J. L. (2010). Ultraviolet radiation and skin cancer. *Int. J. Dermatol.* 49 978–986. 10.1111/j.1365-4632.2010.04474.x 20883261

[B130] Narita M. Nũnez S. Heard E. Narita M. Lin A. W. Hearn S. A. (2003). Rb-mediated heterochromatin formation and silencing of E2F target genes during cellular senescence. *Cell* 113 703–716. 10.1016/S0092-8674(03)00401-X 12809602

[B131] Navarro C. L. Cadiñanos J. De Sandre-Giovannoli A. Bernard R. Courrier S. Boccaccio I. (2005). Loss of ZMPSTE24 (FACE-1) causes autosomal recessive restrictive dermopathy and accumulation of Lamin A precursors. *Hum. Mol. Genet.* 14 1503–1513. 10.1093/hmg/ddi159 15843403

[B132] Niedernhofer L. J. Bohr V. A. Sander M. Kraemer K. H. (2011). Xeroderma pigmentosum and other diseases of human premature aging and DNA repair: molecules to patients. *Mech. Ageing Dev.* 132 340–347. 10.1016/j.mad.2011.06.004 21708183 PMC3474983

[B133] Novelli G. Muchir A. Sangiuolo F. Helbling-Leclerc A. D’Apice M. R. Massart C. (2002). Mandibuloacral dysplasia is caused by a mutation in LMNA-encoding lamin A/C. *Am. J. Hum. Genet.* 71 426–431. 10.1086/341908 12075506 PMC379176

[B134] Nuciforo P. G. Luise C. Capra M. Pelosi G. D’Adda di Fagagna F. (2007). Complex engagement of DNA damage response pathways in human cancer and in lung tumor progression. *Carcinogenesis* 28 2082–2088. 10.1093/carcin/bgm108 17522062

[B135] Ogrodnik M. Miwa S. Tchkonia T. Tiniakos D. Wilson C. L. Lahat A. (2017). Cellular senescence drives age-dependent hepatic steatosis. *Nat. Commun.* 8:15691. 10.1038/ncomms15691 28608850 PMC5474745

[B136] Olovnikov A. M. (1973). A theory of marginotomy. The incomplete copying of template margin in enzymic synthesis of polynucleotides and biological significance of the phenomenon. *J. Theor. Biol.* 41 181–190. 10.1016/0022-5193(73)90198-7 4754905

[B137] Opresko P. L. Shay J. W. (2017). Telomere-associated aging disorders. *Ageing Res. Rev.* 33 52–66. 10.1016/j.arr.2016.05.009 27215853 PMC9926533

[B138] Ortonne J. P. (1990). Pigmentary changes of the ageing skin. *Br. J. Dermatol.* 122(Suppl.), 21–28. 10.1111/j.1365-2133.1990.tb16121.x 2186781

[B139] Oshima J. Sidorova J. M. Monnat R. J. (2017). Werner syndrome: clinical features, pathogenesis and potential therapeutic interventions. *Ageing Res. Rev.* 33 105–114. 10.1016/j.arr.2016.03.002 26993153 PMC5025328

[B140] Passos J. F. Saretzki G. Von Zglinicki T. (2007). DNA damage in telomeres and mitochondria during cellular senescence: is there a connection? *Nucleic Acids Res.* 35 7505–7513. 10.1093/nar/gkm893 17986462 PMC2190715

[B141] Pekovic V. Harborth J. Broers J. L. V. Ramaekers F. C. S. Van Engelen B. Lammens M. (2007). Nucleoplasmic LAP2α-lamin A complexes are required to maintain a proliferative state in human fibroblasts. *J. Cell Biol.* 176 163–172. 10.1083/jcb.200606139 17227891 PMC2063936

[B142] Pittayapruek P. Meephansan J. Prapapan O. Komine M. Ohtsuki M. (2016). Role of matrix metalloproteinases in photoaging and photocarcinogenesis. *Int. J. Mol. Sci.* 17:E868. 10.3390/ijms17060868 27271600 PMC4926402

[B143] Plewig G. Kligman A. M. (1978). Proliferative activity of the sebaceous glands of the aged. *J. Invest. Dermatol.* 70 314–317. 10.1111/1523-1747.ep12543478 649977

[B144] Powell J. B. Dokal I. Carr R. Taibjee S. Cave B. Moss C. (2014). X-linked dyskeratosis congenita presenting in adulthood with photodamaged skin and epiphora. *Clin. Exp. Dermatol.* 39 310–314. 10.1111/ced.12272 24635067

[B145] Quan T. Fisher G. J. (2015). Role of age-associated alterations of the dermal extracellular matrix microenvironment in human skin aging: a mini-review. *Gerontology* 61 427–434. 10.1159/000371708 25660807 PMC4524793

[B146] Quan T. Qin Z. Xia W. Shao Y. Voorhees J. J. Fisher G. J. (2009). Matrix-degrading metalloproteinases in photoaging. *J. Investig. Dermatol. Symp. Proc.* 14 20–24. 10.1038/jidsymp.2009.8 19675548 PMC2909639

[B147] Raddatz G. Hagemann S. Aran D. Söhle J. Kulkarni P. P. Kaderali L. (2013). Aging is associated with highly defined epigenetic changes in the human epidermis. *Epigenetics Chromatin* 6:36. 10.1186/1756-8935-6-36 24279375 PMC3819645

[B148] Ravanat J.-L. Douki T. Cadet J. (2001). Direct and indirect effects of UV radiation on DNA and its components. *J. Photochem. Photobiol. B Biol.* 63 88–102. 10.1016/S1011-1344(01)00206-8 11684456

[B149] Rehman I. Quinn A. G. Healy E. Rees J. L. (1994). High frequency of loss of heterozygosity in actinic keratoses, a usually benign disease. *Lancet* 344 788–789. 10.1016/S0140-6736(94)92343-4 7916075

[B150] Ressler S. Bartkova J. Niederegger H. Bartek J. Scharffetter-Kochanek K. Jansen-Dürr P. (2006). p16INK4A is a robust in vivo biomarker of cellular aging in human skin. *Aging Cell* 5 379–389. 10.1111/j.1474-9726.2006.00231.x 16911562

[B151] Rittié L. Fisher G. J. (2015). Natural and sun-induced aging of human skin. *Cold Spring Harb. Perspect. Med.* 5:a015370. 10.1101/cshperspect.a015370 25561721 PMC4292080

[B152] Rodier F. Coppé J. P. Patil C. K. Hoeijmakers W. A. M. Muñoz D. P. Raza S. R. (2009). Persistent DNA damage signalling triggers senescence-associated inflammatory cytokine secretion. *Nat. Cell Biol.* 11 973–979. 10.1038/ncb1909 19597488 PMC2743561

[B153] Rothblum-Oviatt C. Wright J. Lefton-Greif M. A. McGrath-Morrow S. A. Crawford T. O. Lederman H. M. (2016). Ataxia telangiectasia: a review. *Orphanet J. Rare Dis* 11:159. 10.1186/s13023-016-0543-7 27884168 PMC5123280

[B154] Sadaie M. Salama R. Carroll T. Tomimatsu K. Chandra T. Young A. R. (2013). Redistribution of the Lamin B1 genomic binding profile affects rearrangement of heterochromatic domains and SAHF formation during senescence. *Genes Dev.* 27 1800–1808. 10.1101/gad.217281.113 23964094 PMC3759696

[B155] Scaffidi P. Misteli T. (2006). Lamin A-dependent nuclear defects in human aging. *Science* 312 1059–1063. 10.1126/science.1127168 16645051 PMC1855250

[B156] Schroeder P. Schieke S. M. Morita A. (2006). “Premature skin aging by infrared radiation, tobacco smoke and ozone,” in *Skin Aging*, eds Gilchrest B. A. Krutmann J. (Berlin: Springer), 45–53. 10.1007/3-540-32953-6_5

[B157] Serrano M. Lin A. W. McCurrach M. E. Beach D. Lowe S. W. (1997). Oncogenic RAS provokes premature cell senescence associated with accumulation of p53 and p16(INK4a). *Cell* 88 593–602. 10.1016/S0092-8674(00)81902-9 9054499

[B158] Severino J. Allen R. G. Balin S. Balin A. Cristofalo V. J. (2000). Is beta-galactosidase staining a marker of senescence in vitro and in vivo? *Exp. Cell Res.* 257 162–171. 10.1006/excr.2000.4875 10854064

[B159] Shah P. P. Donahue G. Otte G. L. Capell B. C. Nelson D. M. Cao K. (2013). Lamin B1 depletion in senescent cells triggers large-scale changes in gene expression and the chromatin landscape. *Genes Dev.* 27 1787–1799. 10.1101/gad.223834.113 23934658 PMC3759695

[B160] Sharpless N. E. Sherr C. J. (2015). Forging a signature of in vivo senescence. *Nat. Rev. Cancer* 15 397–408. 10.1038/nrc3960 26105537

[B161] Sherratt M. J. (2013). Age-related tissue stiffening: cause and effect. *Adv. Wound Care* 2 11–17. 10.1089/wound.2011.0328 24527318 PMC3840474

[B162] Shimi T. Butin-Israeli V. Adam S. A. Hamanaka R. B. Goldman A. E. Lucas C. A. (2011). The role of nuclear lamin B1 in cell proliferation and senescence. *Genes Dev.* 25 2579–2593. 10.1101/gad.179515.111 22155925 PMC3248680

[B163] Shumaker D. K. Dechat T. Kohlmaier A. Adam S. A. Bozovsky M. R. Erdos M. R. (2006). Mutant nuclear lamin A leads to progressive alterations of epigenetic control in premature aging. *Proc. Natl. Acad. Sci. U.S.A.* 103 8703–8708. 10.1073/pnas.0602569103 16738054 PMC1472659

[B164] Shuster S. Black M. M. McVitie E. (1975). The influence of age and sex on skin thickness, skin collagen and density. *Br. J. Dermatol.* 93 639–643. 10.1111/j.1365-2133.1975.tb05113.x 1220811

[B165] Siegel J. A. Korgavkar K. Weinstock M. A. (2017). Current perspective on actinic keratosis: a review. *Br. J. Dermatol.* 177 350–358. 10.1111/bjd.14852 27500794

[B166] Solovei I. Wang A. S. Thanisch K. Schmidt C. S. Krebs S. Zwerger M. (2013). LBR and lamin A/C sequentially tether peripheral heterochromatin and inversely regulate differentiation. *Cell* 152 584–598. 10.1016/j.cell.2013.01.009 23374351

[B167] Soufir N. Molès J. P. Vilmer C. Moch C. Verola O. Rivet J. (1999). P16 UV mutations in human skin epithelial tumors. *Oncogene* 18 5477–5481. 10.1038/sj.onc.1202915 10498902

[B168] Stewart C. Burke B. (1987). Teratocarcinoma stem cells and early mouse embryos contain only a single major lamin polypeptide closely resembling lamin B. *Cell* 51 383–392. 10.1016/0092-8674(87)90634-9 3311384

[B169] Sugimoto M. Yamashita R. Ueda M. (2006). Telomere length of the skin in association with chronological aging and photoaging. *J. Dermatol. Sci.* 43 43–47. 10.1016/j.jdermsci.2006.02.004 16524700

[B170] Suram A. Kaplunov J. Patel P. L. Ruan H. Cerutti A. Boccardi V. (2012). Oncogene-induced telomere dysfunction enforces cellular senescence in human cancer precursor lesions. *EMBO J.* 31 2839–2851. 10.1038/emboj.2012.132 22569128 PMC3395091

[B171] Takai H. Smogorzewska A. De Lange T. (2003). DNA damage foci at dysfunctional telomeres. *Curr. Biol.* 13 1549–1556. 10.1016/S0960-9822(03)00542-6 12956959

[B172] Tan W. H. Baris H. Robson C. D. Kimonis V. E. (2005). Cockayne syndrome: the developing phenotype. *Am. J. Med. Genet. A* 135 214–216. 10.1002/ajmg.a.30731 15887300

[B173] Telgenhoff D. Shroot B. (2005). Cellular senescence mechanisms in chronic wound healing. *Cell Death Differ.* 12 695–698. 10.1038/sj.cdd.4401632 15861190

[B174] Tokarsky-Amiel R. Azazmeh N. Helman A. Stein Y. Hassan A. Maly A. (2013). Dynamics of senescent cell formation and retention revealed by p14ARFinduction in the epidermis. *Cancer Res.* 73 2829–2839. 10.1158/0008-5472.CAN-12-3730 23423975

[B175] Tsurumi A. Li W. X. (2012). Global heterochromatin loss: a unifying theory of aging? *Epigenetics* 7 680–688. 10.4161/epi.20540 22647267 PMC3414389

[B176] Vande Berg J. S. Robson M. C. (2003). Arresting cell cycles and the effect on wound healing. *Surg. Clin. North Am.* 83 509–520. 10.1016/S0039-6109(02)00195-0 12822722

[B177] Vande Berg J. S. Rose M. A. Haywood-Reid P. L. Rudolph R. Payne W. G. Robson M. C. (2005). Cultured pressure ulcer fibroblasts show replicative senescence with elevated production of plasmin, plasminogen activator inhibitor-1, and transforming growth factor-β1. *Wound Repair Regen.* 13 76–83. 10.1111/j.1067-1927.2005.130110.x 15659039

[B178] Varani J. Dame M. K. Rittie L. Fligiel S. E. G. Kang S. Fisher G. J. (2006). Decreased collagen production in chronologically aged skin. *Am. J. Pathol.* 168 1861–1868. 10.2353/ajpath.2006.051302 16723701 PMC1606623

[B179] Varani J. Schuger L. Dame M. K. Leonard C. Fligiel S. E. G. Kang S. (2004). Reduced fibroblast interaction with intact collagen as a mechanism for depressed collagen in synthesis in photodamaged skin. *J. Invest. Dermatol.* 122 1471–1479. 10.1111/j.0022-202X.2004.22614.x 15175039

[B180] Vidak S. Foisner R. (2016). Molecular insights into the premature aging disease progeria. *Histochem. Cell Biol.* 145 401–417. 10.1007/s00418-016-1411-1 26847180 PMC4796323

[B181] Vierkötter A. Schikowski T. Ranft U. Sugiri D. Matsui M. Krämer U. (2010). Airborne particle exposure and extrinsic skin aging. *J. Invest. Dermatol.* 130 2719–2726. 10.1038/jid.2010.204 20664556

[B182] Vijg J. Suh Y. (2013). Genome Instability and Aging. *Annu. Rev. Physiol.* 75 645–668. 10.1146/annurev-physiol-030212-183715 23398157

[B183] Waaijer M. Gunn D. A. Heemst D. Van Slagboom P. E. John M. Dirks R. W. (2018). Do senescence markers correlate in vitro and in situ within individual human donors? *Aging* 10 278–289. 10.18632/aging.101389 29500330 PMC5842854

[B184] Waaijer M. Parish W. E. Strongitharm B. H. van Heemst D. Slagboom P. E. de Craen A. J. M. (2012). The number of p16INK4a positive cells in human skin reflects biological age. *Aging Cell* 11 722–725. 10.1111/j.1474-9726.2012.00837.x 22612594 PMC3539756

[B185] Wajapeyee N. Serra R. W. Zhu X. Mahalingam M. Green M. R. (2008). Oncogenic BRAF induces senescence and apoptosis through pathways mediated by the secreted protein IGFBP7. *Cell* 132 363–374. 10.1016/j.cell.2007.12.032 18267069 PMC2266096

[B186] Wang A. S. Ong P. F. Chojnowski A. Clavel C. Dreesen O. (2017). Loss of lamin B1 is a biomarker to quantify cellular senescence in photoaged skin. *Sci. Rep.* 7:15678. 10.1038/s41598-017-15901-9 29142250 PMC5688158

[B187] Watson J. D. (1972). Origin of concatemeric T7DNA. *Nat. New Biol.* 239 197–201. 10.1038/newbio239197a0 4507727

[B188] Watson R. E. B. Gibbs N. K. Griffiths C. E. M. Sherratt M. J. (2014). Damage to skin extracellular matrix induced by UV exposure. *Antioxid. Redox Signal.* 21 1063–1077. 10.1089/ars.2013.5653 24124905

[B189] Wheaton K. Campuzano D. Ma W. Sheinis M. Ho B. Brown G. W. (2017). Progerin-induced replication stress facilitates premature senescence in hutchinson-gilford progeria syndrome. *Mol. Cell. Biol.* 37 e659-16. 10.1128/MCB.00659-16 28483909 PMC5492170

[B190] Wiley C. D. Velarde M. C. Lecot P. Liu S. Sarnoski E. A. Freund A. (2016). Mitochondrial dysfunction induces senescence with a distinct secretory phenotype. *Cell Metab.* 23 303–314. 10.1016/j.cmet.2015.11.011 26686024 PMC4749409

[B191] Wlaschek M. Tantcheva-Poór I. Naderi L. Ma W. Schneider L. A. Razi-Wolf Z. (2001). Solar UV irradiation and dermal photoaging. *J. Photochem. Photobiol. B Biol.* 63 41–51. 10.1016/S1011-1344(01)00201-9 11684450

[B192] Wood A. M. Danielsen J. M. R. Lucas C. A. Rice E. L. Scalzo D. Shimi T. (2015). TRF2 and lamin A/C interact to facilitate the functional organization of chromosome ends. *Nat. Commun.* 5:5467. 10.1038/ncomms6467 25399868 PMC4235626

[B193] Xu G. Snellman E. Bykov V. J. Jansen C. T. Hemminki K. (2000). Effect of age on the formation and repair of UV photoproducts in human skin in situ. *Mutat. Res.* 459 195–202. 10.1016/S0921-8777(99)00069-5 10812331

[B194] Xu M. Palmer A. K. Ding H. Weivoda M. M. Pirtskhalava T. White T. A. (2015). Targeting senescent cells enhances adipogenesis and metabolic function in old age. *elife* 4:e12997. 10.7554/eLife.12997 26687007 PMC4758946

[B195] Yamada M. Udono M. U. Hori M. Hirose R. Sato S. Mori T. (2006). Aged human skin removes UVB-induced pyrimidine dimers from the epidermis more slowly than younger adult skin in vivo. *Arch. Dermatol. Res.* 297 294–302. 10.1007/s00403-005-0618-0 16328344

[B196] Yamada S. Maruyama I. (2007). HMGB1, a novel inflammatory cytokine. *Clin. Chim. Acta* 375 36–42. 10.1016/j.cca.2006.07.019 16979611

[B197] Yosef R. Pilpel N. Tokarsky-Amiel R. Biran A. Ovadya Y. Cohen S. (2016). Directed elimination of senescent cells by inhibition of BCL-W and BCL-XL. *Nat. Commun.* 7:11190. 10.1038/ncomms11190 27048913 PMC4823827

[B198] You Y. H. Lee D. H. Yoon J. H. Nakajima S. Yasui A. Pfeifer G. P. (2001). Cyclobutane pyrimidine dimers are responsible for the vast majority of mutations induced by UVB irradiation in mammalian cells. *J. Biol. Chem.* 276 44688–44694. 10.1074/jbc.M107696200 11572873

[B199] Yu C. E. Oshima J. Fu Y. H. Wijsman E. M. Hisama F. Alisch R. (1996). Positional cloning of the Werner’s syndrome gene. *Science* 72 258–262. 10.1126/science.272.5259.258 8602509

[B200] Zhang J. Lian Q. Zhu G. Zhou F. Sui L. Tan C. (2011). A human iPSC model of hutchinson gilford progeria reveals vascular smooth muscle and mesenchymal stem cell defects. *Cell Stem Cell* 8 31–45. 10.1016/j.stem.2010.12.002 21185252

[B201] Zhang W. Li J. Suzuki K. Qu J. Wang P. Zhou J. (2015). A Werner syndrome stem cell model unveils heterochromatin alterations as a driver of human aging. *Science* 348 1160–1163. 10.1126/science.aaa1356 25931448 PMC4494668

[B202] Zhu Y. Tchkonia T. Fuhrmann-Stroissnigg H. Dai H. M. Ling Y. Y. Stout M. B. (2016). Identification of a novel senolytic agent, navitoclax, targeting the Bcl-2 family of anti-apoptotic factors. *Aging Cell* 15 428–435. 10.1111/acel.12445 26711051 PMC4854923

[B203] Zindy F. Quelle D. E. Roussel M. F. Sherr C. J. (1997). Expression of the p16INK4a tumor suppressor versus other INK4 family members during mouse development and aging. *Oncogene* 15 203–211. 10.1038/sj.onc.1201178 9244355

[B204] Zou L. (2007). Single- and double-stranded DNA: building a trigger of ATR-mediated DNA damage response. *Genes Dev.* 21 879–885. 10.1101/gad.1550307 17437994

[B205] Zouboulis C. C. Boschnakow A. (2001). Chronological ageing and photoageing of the human sebaceous gland. *Clin. Exp. Dermatol.* 26 600–607. 10.1046/j.1365-2230.2001.00894.x 11696064

